# Response Surface Methodology (RSM) Optimization of Electromagnetic Interference (EMI) Shielding Effectiveness in Polymer Nanocomposites with Irradiated Hybrid Carbon Nanostructures

**DOI:** 10.3390/polym18162024

**Published:** 2026-08-21

**Authors:** Anita Grozdanov, Stefan Kuvendziev, Iva Dimitrievska, Mirko Marinkovski, Martin Stojchevski, Andrea Petanova, Perica Paunović, Duska Kleut, Svetlana Jovanović

**Affiliations:** 1 Faculty of Technology and Metallurgy, Ss. Cyril and Methodius University in Skopje, Rugjer Boshkovikj 16, 1000 Skopje, North Macedonia; anita@tmf.ukim.edu.mk (A.G.); stefan@tmf.ukim.edu.mk (S.K.); mirko@tmf.ukim.edu.mk (M.M.); martin@tmf.ukim.edu.mk (M.S.); andrea.petan@gmail.com (A.P.); pericap@tmf.ukim.edu.mk (P.P.); 2 Vinča Institute of Nuclear Sciences—National Institute of the Republic of Serbia, University of Belgrade, P.O. Box 522, 11000 Belgrade, Serbia; duska@vin.bg.ac.rs (D.K.); svetlanajovanovic@vin.bg.ac.rs (S.J.)

**Keywords:** EMI shielding, graphene–carbon nanotube, PMMA composites, absorption-dominant shielding, response surface methodology, S-band

## Abstract

In recent decades, due to the rapid development and application of wireless communication, flexible electronics, and smart devices, electromagnetic interference (EMI) and radiation pollution have been intensified, creating an urgent demand for efficient EMI shielding materials. Carbon nanostructures such as graphene and carbon nanotubes are considered promising candidates due to their excellent properties, such as high electrical conductivity, low density, large specific surface area, and flexibility. This work reports our recent results in the design and testing of polymer nanocomposites with irradiated hybrid carbon nanostructure (graphene/multi-walled carbon nanotubes) used as EMI shielding materials. Five representative composites with varying filler loadings (AH of 15% and AM1 of 20 wt%), thicknesses (0.208–0.48 mm), and e-beam irradiation doses (from 50 to 400 kGy) were systematically characterized using SEM, FTIR, TGA/DSC, and vector network analyzer (VNA) measurements in the S-band (2.65–3.90 GHz). The effects of different e-beam irradiation doses and hybrid carbon contents on conductive network construction, interface engineering, and porous or layered structures on EMI shielding performance are discussed. Experimental results show that all studied composites exhibited strong absorption-dominant behavior (SE_A_), while the multiple reflection component (SE_M_) was found to be negligible. Both filler loading and sample thickness significantly enhanced shielding performance, with a pronounced synergistic interaction observed between these parameters. A quadratic Response Surface Methodology (RSM) model was developed to correlate the total shielding effectiveness (SE_T_) with thickness and filler content, yielding high predictive accuracy (*R*^2^ > 0.96). The model enables efficient optimization of composite design for targeted shielding levels.

## 1. Introduction

The rapid proliferation of electronic devices, wireless communication systems, and high-frequency technologies such as 5G and radar has led to a significant increase in electromagnetic interference (EMI). Uncontrolled electromagnetic waves not only degrade the performance and reliability of sensitive electronic equipment but also raise concerns regarding human health and information security [1,2]. In addition, the increasing numbers of digital networks and wireless control systems generate electromagnetic noise (EMN) or destroy electromagnetic compatibility (EMC). Hence, all electronic devices need EMI shielding systems as the world relies on electronic components. Consequently, the development of high-performance EMI shielding materials has become a critical requirement across aerospace, defense, automotive, and consumer electronics industries.

Traditional metal-based shields, while effective, suffer from several limitations, including high density, susceptibility to corrosion, and reflection-dominant shielding mechanisms that can cause secondary interference. These drawbacks have driven extensive research toward lightweight, flexible, and absorption-dominant polymer composites filled with conductive nanomaterials. Polymer/carbon nanostructures (graphene, carbon nanotubes) represent one of the best configurations to offer acceptable absorption-based EMI shielding due to their lower weight and hydrophobicity, high electrical conductivity, and the formation of a strong three-dimensional interconnected electrically conductive network with porous microstructures. This composite architecture provides a microstructure that allows electromagnetic waves to enter the material through impedance matching. The nature of EMI shielding phenomena in carbon-based polymer nanocomposites has been described and discussed in several review papers [2,3,4,5]. Among various fillers, carbon-based materials such as graphene and carbon nanotubes (CNTs) have attracted particular attention due to their exceptional electrical conductivity, high aspect ratio, and large specific surface area, which facilitate the formation of conductive networks and promote dielectric loss [5,6]. Hybrid fillers combining graphene flakes and carbon nanotubes (G-CNT) offer synergistic advantages over single-filler systems. The two-dimensional graphene sheets provide large interfacial areas for polarization, while the one-dimensional CNTs bridge graphene sheets, enhancing the overall conductive network and reducing the percolation threshold. Furthermore, post-processing techniques such as irradiation have shown promise in improving filler dispersion, matrix–filler interfacial bonding, and the overall electrical properties of polymer composites. Jovanović et al. [7] reviewed studies investigating various graphene-based composites as potential EMI shielding materials. Specifically, they provided an overview of polymer nanocomposites reinforced with graphene and silver nanowires due to their high EMI shielding efficiency, low production cost, and favorable mechanical properties. Pavlou and co-workers studied the EMI behavior of graphene/PMMA nanolaminates in the THz region [8]. They produced nanolaminates with a fixed graphene content of 0.33 vol.% using an iterative ‘lift-off/float-on’ process. It was found that these thin laminate materials show high EMI shielding efficiency, reaching 60 dB at a thickness of only 33 μm, and an absolute EMI shielding effectiveness close to 3 × 10^5^ dB cm^2^ g^−1^, which is among the highest values for non-metallic materials.

Poly(methyl methacrylate) (PMMA), as a lightweight, nontoxic, and environmentally friendly material that is ideal for the preparation of polymer composites, is a particularly attractive polymer matrix for EMI shielding applications owing to its excellent optical transparency, mechanical strength, dimensional stability, and ease of processing. However, the EMI shielding performance of G-CNT/PMMA composites is strongly influenced by multiple parameters, including filler loading, sample thickness, and processing conditions. Understanding and optimizing these interdependent variables is essential for designing materials with tailored shielding effectiveness.

Anderson and his team [9] published a review in which they elaborated on accurate modelling techniques based on the underlying micromechanics, such as percolation, electron tunneling, agglomeration, imperfect interfaces, frequency-dependent nanocapacitance, and electron hopping. They pointed out that numerical solutions are essential in the modeling of electromagnetic waves and that all advanced modern simulation software currently relies on numerical solutions, such as the Finite Element Method (FEM), Finite Volume Method (FVM), Finite Difference Time Domain (FDTD), and Finite Integration Technique (FIT). They reported a comparison of each of these methods regarding their application to electromagnetic propagation problems. Anderson et al. [9] also worked on designing a model for predicting the electromagnetic properties of nanocomposites based on the pre-determined properties of the polymer matrix and the carbon-based filler, directing their focus specifically to a graphene nanoplatelet (GnP)/epoxy system. The research of Xia and coworkers was focused on the multi-objective optimization of highly efficient EMI shielding in porous graphene-reinforced nanocomposites [10]. First, a two-scale electromagnetic constitutive model of EMI shielding effectiveness (SE) and cost was established through the effective-medium approximation with tunneling and Maxwell–Wagner–Sillars polarization effects. Then, an NSGA-II-based multi-objective optimization was developed for high EMI SE and low cost with the assistance of crowding distance and an elite strategy.

While many studies report experimental EMI shielding data, relatively few have employed systematic statistical modeling approaches such as Response Surface Methodology (RSM) to quantify the effects of key processing and design parameters. Such models are invaluable for reducing experimental workload and enabling predictive design of composites for specific shielding targets.

The present study investigates the EMI shielding performance of hybrid G-CNT-reinforced PMMA composites in the S-band frequency range (2.65–3.90 GHz). Five representative samples with varying filler loadings (15–20 wt%), thicknesses (0.208–0.48 mm), and irradiation treatments (from 50 to 400 kGy) were fabricated and characterized using vector network analyzer (VNA) measurements. An additional contribution of this paper is that the same samples integrate three factors—irradiation treatment, various filler loadings, and film thickness. The shielding mechanisms (reflection, absorption, and multiple reflections) were thoroughly analyzed, and a quadratic Response Surface Model was developed to correlate shielding effectiveness with material design parameters. The primary objectives of this work are to evaluate the influence of filler loading and sample thickness on total shielding effectiveness (SE_T_) and its components, to demonstrate the absorption-dominant behavior of the developed composites, and to establish a predictive RSM model for efficient material optimization.

## 2. Materials and Methods

### 2.1. Materials and Composite Preparation

Poly(methyl methacrylate) (PMMA) with a molecular weight of 150,000 g/mol was used as the polymer matrix for the prepared nanocomposites. A hybrid graphene–carbon nanotube (G-CNT) material, obtained from a supplier in China, was utilized as the conductive filler. All chemicals and dimethylformamide (DMF) solvent were of analytical grade and used as received. The solution-processing method was selected because it provides a rapid, uniform, and straightforward approach to fabricating functional materials via a simple, cost-effective, and reproducible technique. The sample designations used throughout this work are as follows: four samples containing 15 wt% hybrid filler (denoted as AH) irradiated with doses ranging from 50 to 400 kGy, and one sample containing 20 wt% hybrid filler (denoted as AM1) irradiated at 50 kGy.

The final film thicknesses were controlled by adjusting the casting parameters (solution volume) and the dimensions of the Petri dishes, resulting in samples with thicknesses ranging from 0.208 mm to 0.48 mm. A pure PMMA film was also prepared following the same procedure to serve as a reference.

### 2.2. Characterization of Nanocomposites

The hybrid G-MWCNT nanostructures were irradiated with an e-beam using a linear accelerator (ELU-6, Eksma, Vilnius, Lithuania) across a dose range of 50, 100, 200, and 400 kGy determined by calibrated polymer film dosimetry (B3000, B3 WINdose Dosimeters, GEX Corporation, Palm City, FL, USA).

TGA and DSC measurements were performed on representative samples (~20 mg) using a simultaneous TGA/DSC thermal analyzer (Themis One+, Setaram, Sophia Antipolis, France) under a nitrogen atmosphere, at a heating rate of 10 °C min^−1^ over a temperature range of 30–800 °C.

FTIR-ATR spectra were recorded in the 3500–400 cm^−1^ wavenumber range using a Varian 660 FTIR spectrometer (Agilent Technologies, Santa Clara, CA, USA).

The microstructure and morphology of the pristine and irradiated G-MWCNT carbon hybrid nanostructures and composite films were analyzed using scanning electron microscopy (SEM) (model JT-FFX, JEOL, Tokyo, Japan). Transmission electron microscopy (TEM) observations of the fabricated samples were carried out using an FEI Tecnai G2 Spirit TWIN transmission electron microscope equipped with a LaB6 cathode.

The electrical conductivity of the films was determined from electrical resistivity measurements performed at room temperature using a four-point probe instrument (Jandel RM3000, Jandel Engineering Limited, Leighton Buzzard, UK) [11]. The measured resistivity values were converted from Ω·cm to Ω·m, after which the electrical conductivity (S/m) was calculated as the reciprocal of the resistivity. The film samples were tested directly without additional sample preparation, and the reported values represent the average of three measurements.

### 2.3. Electromagnetic Characterization

A P9370A vector network analyzer (VNA) (Keysight, Santa Rosa, CA, USA) was used to perform the EMI SE measurements. The dimensions of the samples matched the internal cross-section of the waveguide adapters used for the electromagnetic shielding measurements. The transmission and reflection coefficients were measured in the S-band frequency range (2.60–3.95 GHz) using WR-284 waveguide adapters (Pasternack, Irvine, CA, USA). The selection of this specific frequency range is highly relevant for modern telecommunications, as the S-band hosts critical infrastructure for wireless devices, including sub-6 GHz 5G mobile networks (3.3–3.8 GHz) and satellite communications.

**Figure 1:**
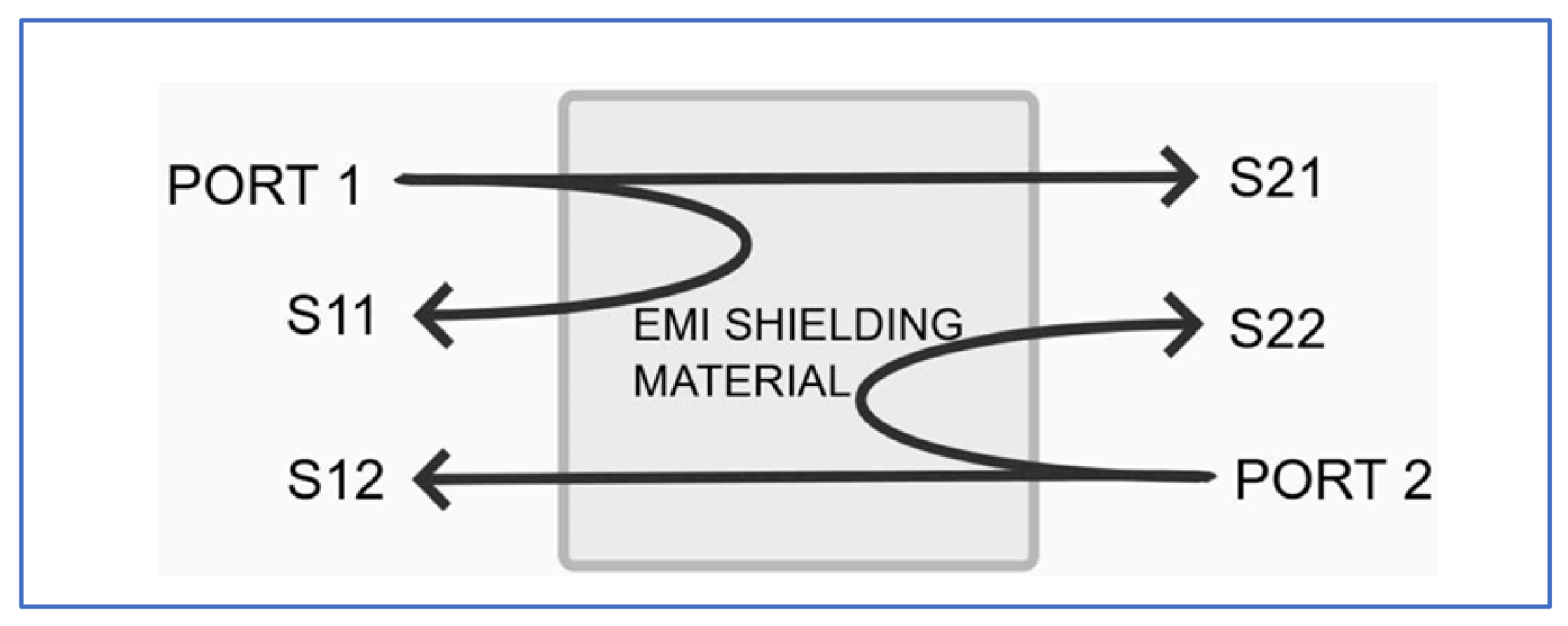
Complex of scattering parameters registered for the tested EMI material from a two-port VNA.

The waveguide adapters were connected to Ports 1 and 2 of the VNA using RF coaxial cables (Figure 1). The samples were clamped between the two waveguide adapters, and the scattering parameters (S11 and S21) were collected. All measurements were conducted at room temperature. The scattering parameters were recorded in both magnitude (dB) and phase (degrees).

The EMI shielding effectiveness parameters were calculated as follows:

(1) 1 = R + T + A

(2) $$R={\;\left|S_{11}\right|}^{2}={\left|S_{22}\right|}^{2}$$

(3) $$T={\left|S_{12}\right|}^{2}={\left|S_{21}\right|}^{2}$$

(4) $${SE}_{R}=-10\log_{10}\left(1-{\left|S_{11}\right|}^{2}\right)$$

(5) $${SE}_{A}=-10\log_{10}\left(\frac{{\left|S_{21}\right|}^{2}}{1-{\left|S_{11}\right|}^{2}}\right)$$

(6) $${SE}_{T}={SE}_{R}+{SE}_{A}+{SE}_{M}$$

(7) $${SE}_{T}\;\approx \;{SE}_{R}+{SE}_{A}=-10\log_{10}\left({\left|S_{21}\right|}^{2}\right)$$

where *R*, *T*, and *A* represent the reflected, transmitted, and absorbed power coefficients, respectively. The terms |S11|, |S12|, |S21| and |S22| are the linear magnitudes of the scattering parameters converted from their respective dB values. Furthermore, SE_A_, SE_R_, SE_M_, and SE_T_ represent the shielding effectiveness associated with absorption, reflection, multiple internal reflections, and total shielding effectiveness, respectively [12].

### 2.4. Statistical Modeling and Data Analysis

Response Surface Methodology (RSM) was employed to model the relationship between input variables (filler loading and sample thickness) and the response variable (average *SE_T_*). A quadratic polynomial model was fitted using the experimental data from the five representative samples:

(8) $${SE}_{T}\;={\;\beta }_{0}\;+\;\beta_{1}d\;+\;\beta_{2}W\;+\;\beta_{3}I\;+\;\beta_{4}d^{2}\;+\;\beta_{5}dW\;+\;\beta_{6}dI\;+\;\beta_{7}W^{2}\;+\;\beta_{8}WI\;+\;\beta_{9}I^{2}\;$$

where *d* is the sample thickness (mm), *W* is the G-CNT loading (wt%), *I* is the irradiation dose (kGy), and *β*_0_–*β*_9_ are the model coefficients.

The optimization objective of this RSM study was to maximize the total shielding effectiveness (SE_T_) within the defined experimental boundaries (0.208 ≤ d ≤ 0.48 mm; 15 ≤ W ≤ 20 wt%; 50 ≤ I ≤ 400 kGy). Rather than seeking a single global maximum, the model in Equation (8) was used to identify optimal design regions, visualized as 3D surfaces later in this manuscript, where synergistic interactions between parameters allow for flexible material tailoring to meet specific shielding requirements.

Model fitting, analysis of variance (ANOVA), and three-dimensional (3D) response surface generation were performed using Statgraphics Centurion XV.II (with libraries such as Pandas, NumPy, and Statsmodels/Plotly). All data processing, including conversion of S-parameters and calculation of shielding components, was carried out using custom scripts to ensure accuracy and traceability.

The correlation between the independent material design parameters and the resulting shielding performance was established using least-squares regression within a Response Surface Methodology (RSM) framework. A second-order (quadratic) polynomial model—incorporating linear effects, quadratic terms, and two-factor interaction terms—was fitted to the experimental data to provide a robust balance between fit quality and physical interpretability. The corresponding regression equation, which describes the functional dependence of the Total Shielding Effectiveness (SE_T_) on the investigated composite parameters, is expressed in terms of the actual variables.

## 3. Results and Discussion

### 3.1. Structural and Thermal Analysis of PMMA/G-MWCNT Nanocomposites

**Figure 2:**
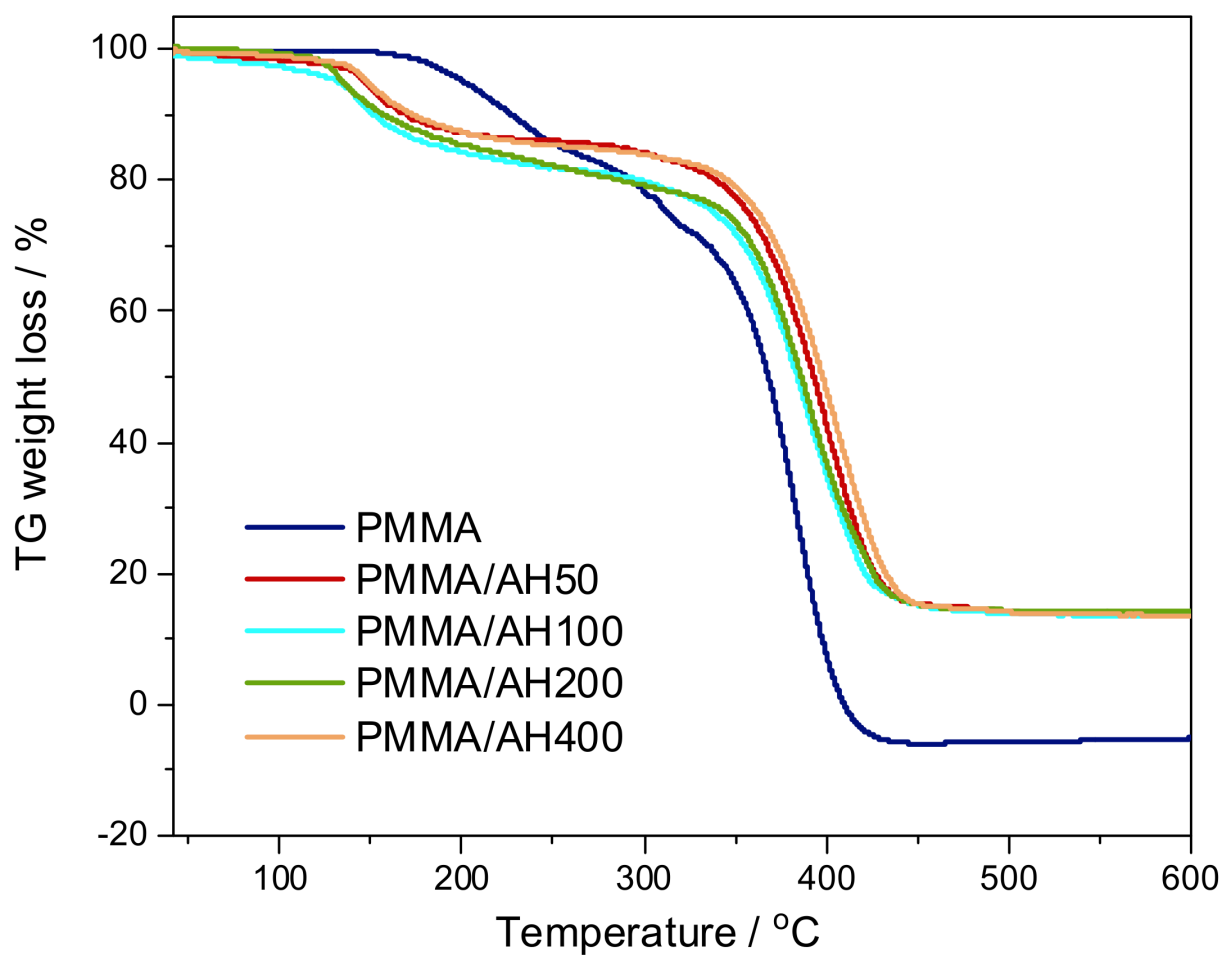
TGA thermograms of the PMMA/AH hybrid G-MWCNT nanocomposite films.

**Figure 3:**
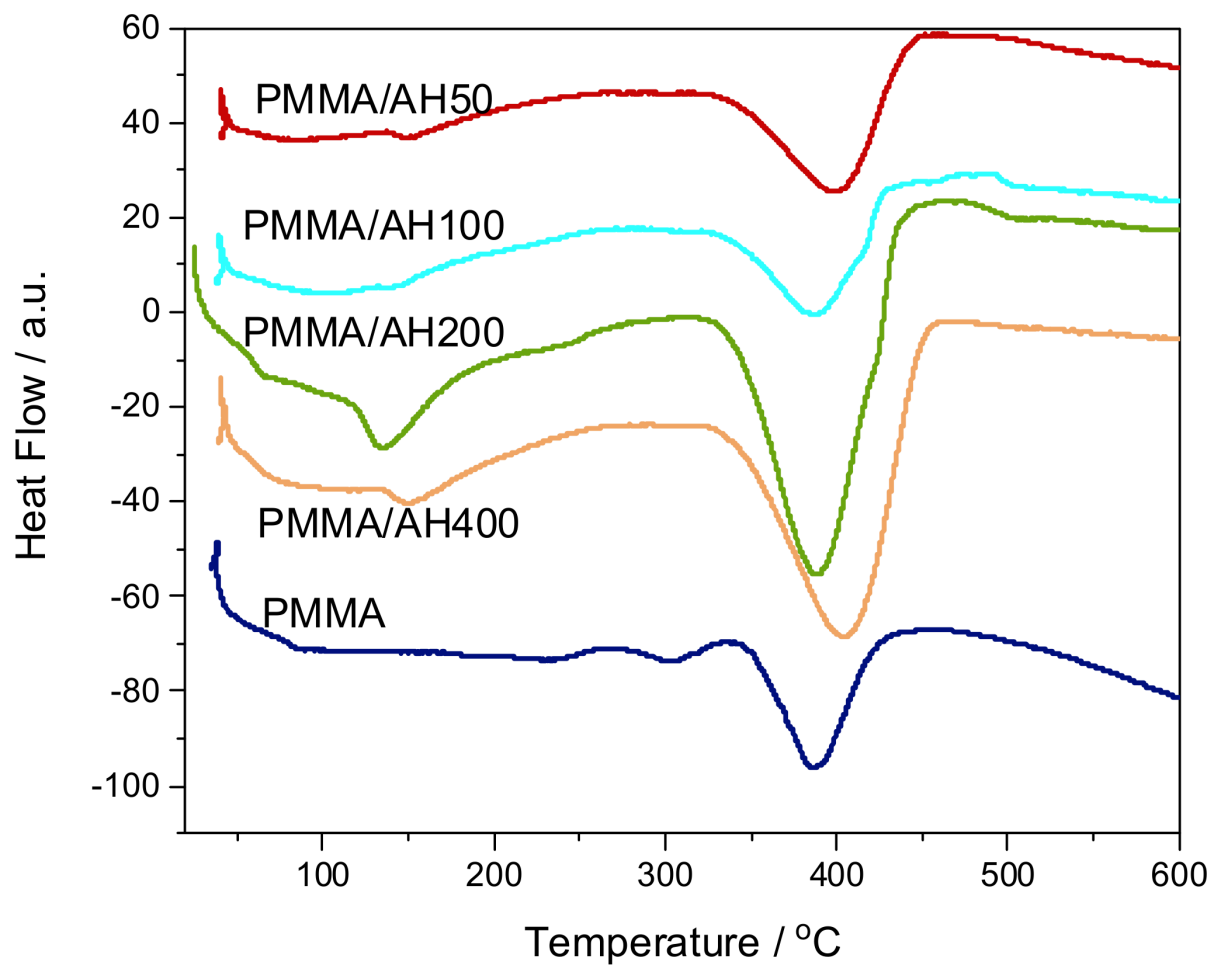
DSC thermograms of the PMMA/AH hybrid nanocomposite films.

Typical TGA thermograms of the studied hybrid G-MWCNT nanostructures are shown in Figure 2, while the obtained DSC curves are shown in Figure 3. It is evident that with the incorporation of G-MWCNT nanoparticles into the polymer matrix, the degradation temperature shifted slightly to higher values, indicating improved thermal stability and good dispersion of the hybrid nanostructures within the PMMA polymer chains. All tested samples exhibited a minor initial weight loss (between 1% and 4%) below 150 °C, which is usually related to the desorption of physically adsorbed moisture trapped between the high-surface-area layers.

The same tendency was obtained via DSC analysis, where the peak temperature was shifted to higher values. Furthermore, increasing the irradiation dose of the G-MWCNTs shifted the glass transition temperature (*T*g) of the polymer matrix to higher values. This shift reflects restricted polymer chain mobility within the nanocomposites reinforced with G-MWCNTs irradiated at the higher dose of 400 kGy.

**Figure 4:**
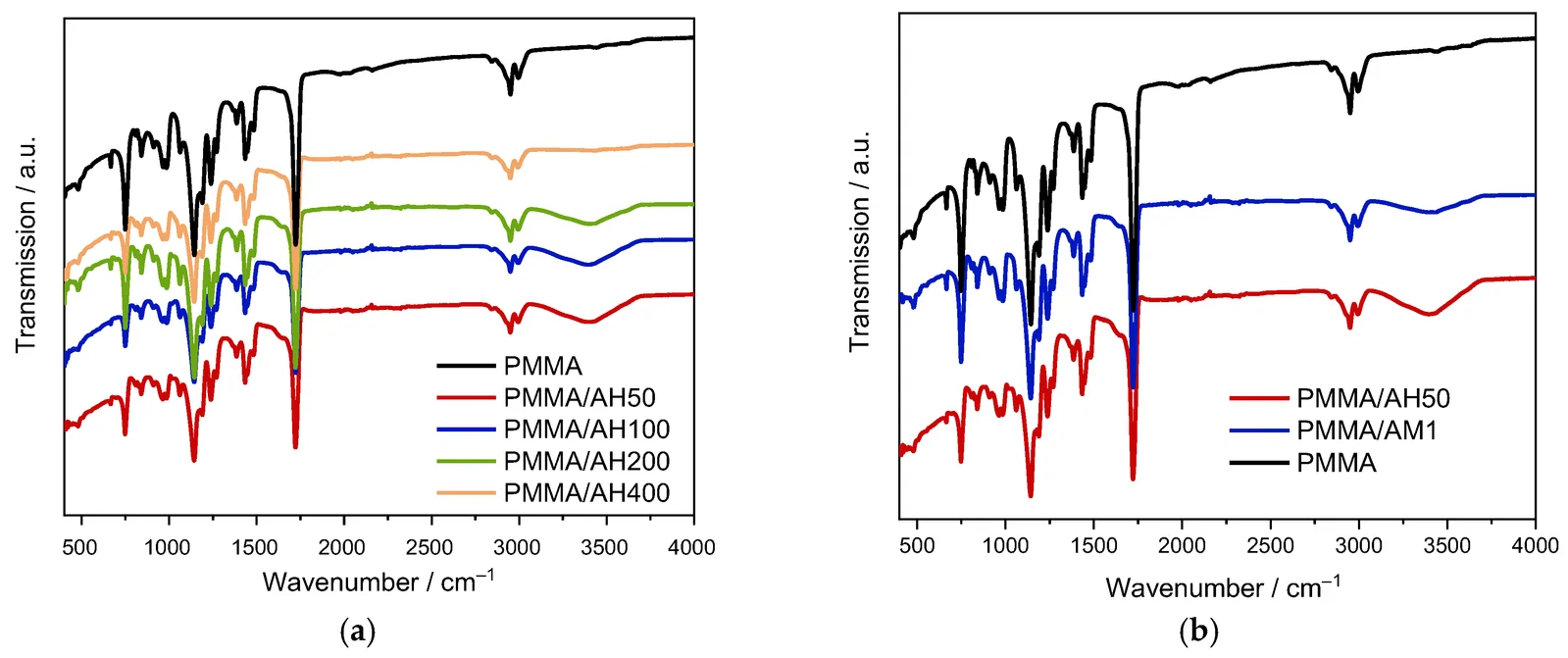
FTIR-ATR spectra of the studied materials: (**a**) pristine PMMA and PMMA/AH hybrid nanocomposites subjected to varying e-beam irradiation doses (50 to 400 kGy) and (**b**) comparison of pristine PMMA with PMMA/AH50 (15 wt% filler) and PMMA/AM1 (20 wt% filler) nanocomposites. Note: The spectra are vertically offset in a stacked view for visual clarity.

The FTIR spectra of the pristine PMMA and the prepared PMMA/hybrid nanocomposites are presented in Figure 4. The spectra suggest that the primary chemical structure of the PMMA matrix is preserved, indicating that the PMMA polymer chains and the G-MWCNT nanoparticles interact non-covalently, as evidenced by the absence of any new chemical bands. The main characteristic peaks of the PMMA matrix dominate the spectra across all studied configurations. A broad band at 3420 cm^−1^ is assigned to the hydroxyl group (O–H) stretching vibrations, which is clearly visible in the lower-dose nanocomposites (PMMA/AH50 and PMMA/AH100) but attenuates significantly at higher irradiation doses. Strong peaks corresponding to the C–H stretching vibrations of the methyl (–CH_3_) and methylene (–CH_2_–) groups appear at 2996 cm^−1^ and 2857 cm^−1^, respectively, while the corresponding C–H bending and deformation vibration peaks of these aliphatic groups are observed between 1447 cm^−1^ and 1338 cm^−1^ [8]. A highly intense, sharp peak at 1723 cm^−1^ is attributed to the carbonyl group (C=O) stretching of the ester functional group. The characteristic multi-peaked region corresponding to the asymmetric and symmetric C–O–C stretching vibrations of the ester linkage in PMMA is resolved at 1240 cm^−1^ and 1150 cm^−1^. In the lower wavenumber fingerprint region, distinct peaks appearing at 985 cm^−1^, 747 cm^−1^, and 483 cm^−1^ correspond to the polymer backbone skeletal modes and out-of-plane C–H bending bands [9,13]. Additionally, the skeletal ring (C=C) stretching vibration of the sp^2^ carbon domain, characteristic of the graphene-based sheets, is discernible as a subtle shoulder near 1604 cm^−1^ adjacent to the dominant carbonyl peak [14,15].

The measured electrical conductivity data for the obtained nanocomposites are shown in Table 1. All nanocomposite films have a thickness of 0.208 to 0.280 mm. Compared to the neat PMMA matrix, all PMMA-based nanocomposite films filled with the irradiated carbon hybrid nanostructures exhibit a remarkable increase in electrical conductivity. Generally, according to Pavlou and his team [8], the electrical conductivity of graphene/PMMA-based nanocomposite samples depends on and results from issues in obtaining uniform dispersion of the graphene filler inside the polymer matrix without aggregation [8]. An increase in electrical conductivity was also observed with increasing irradiation doses. This drop is likely attributed to severe structural degradation of the carbon network under excessive radiation. This induced structural disorder within the hybrid nanostructures subsequently leads to a lower total EMI shielding effectiveness (SE_T_) in the corresponding nanocomposites.

**Figure 5:**
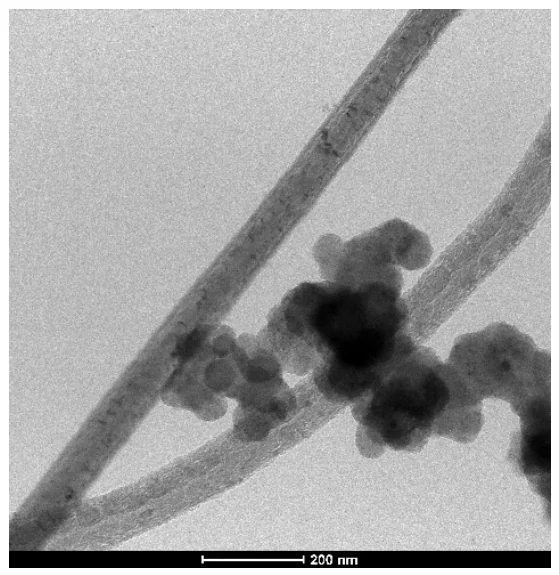
TEM of the G-MWCNT carbon hybrid nanostructure.

**Figure 6:**
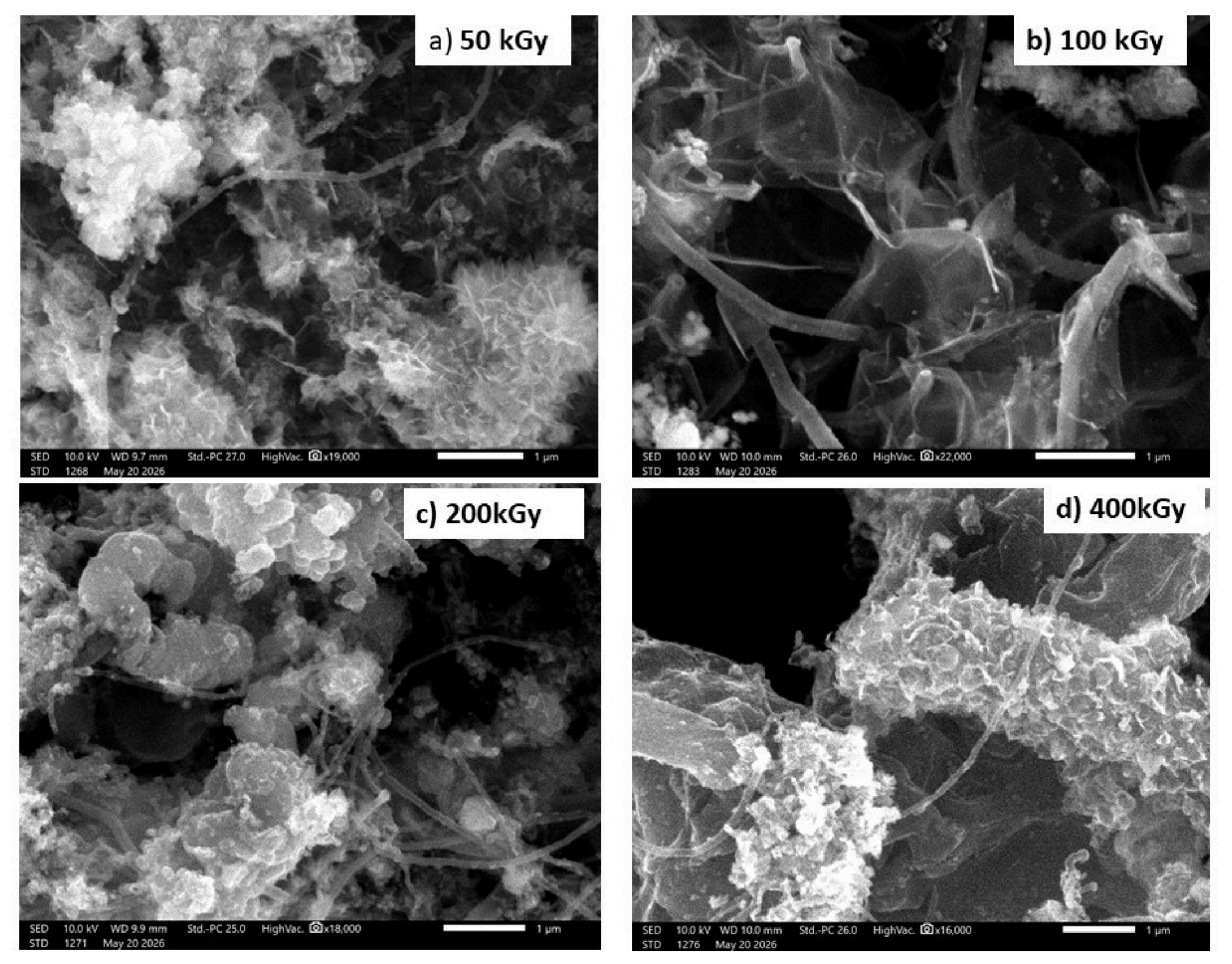
SEM micrographs of irradiated G/MWCNT carbon hybrid nanostructures: (**a**) AH 50kGy, ×19,000; (**b**) AH 100 kGy, ×22,000; (**c**) AH 200 kGy, ×18,000; and (**d**) AH 400 kGy, ×16,000.

**Figure 7:**
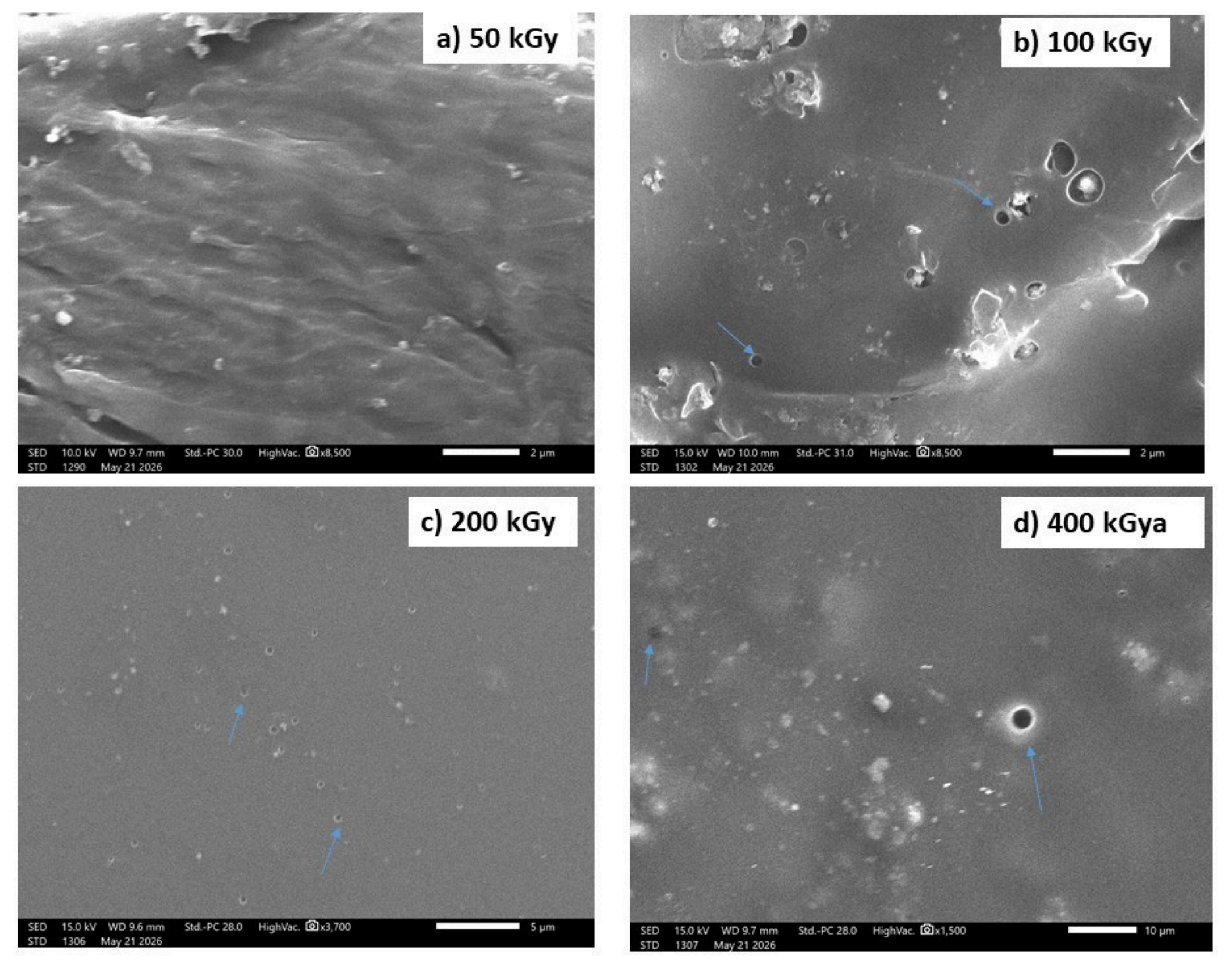
SEM microphotographs of PMMA-polymer-based nanocomposites with irradiated G/MWCNT carbon hybrid nanostructures: (**a**) PMMA/AH50kGy, ×8500; (**b**) PMMA/AH100 kGy, ×8500; (**c**) PMMA/AH200kGy, ×3700; and (**d**) PMMA/AH400 kGy, ×1500.

The hybrid G-MWCNT nanostructures were treated with various irradiation doses (50, 100, 200, and 400 kGy) to activate the carbon nanoparticles and to enhance the conductive network within the PMMA polymer matrix. The morphology of the hybrid nanostructures was studied using TEM and SEM. A typical TEM micrograph of the G-MWCNT hybrid is shown in Figure 5. The SEM micrographs of the irradiated G-MWCNT nanostructures are presented in Figure 6, while the microstructures of the corresponding PMMA-based nanocomposite films are shown in Figure 7.

The SEM images confirm the presence of graphene flakes and carbon nanotubes. Namely, SEM micrographs of irradiated G/MWCNT carbon hybrid nanostructures confirmed the morphological changes due to the higher irradiation dose; higher separation of the graphene flakes was observed in the samples irradiated with 200 (Figure 6c) and 400 kGy (Figure 6d). At higher irradiation doses, the graphene flakes appear highly exfoliated. The obtained microstructures significantly boost the EMI-SE of the composites. The neat and low-dose polymer nanocomposites exhibit a relatively smooth cross-sectional surface, except for the nanocomposite filled with the G-MWCNT irradiated with 50 kGy, as shown in Figure 7a. The G-MWCNT carbon hybrid nanoparticles are well distributed and successfully incorporated within the polymer matrix. However, within the nanocomposites containing G-MWCNTs irradiated at higher doses (100, 200, and 400 kGy), numerous nanopores (shown with arrows in Figure 7b–d) with an average diameter of 250–290 nm were formed. These nanopores are expected to influence and subsequently decrease the EMI SE of the studied nanocomposites. According to the literature, porous structures bring more interfaces and defects, which are conducive to interface polarization and dipole polarization, and enhance the attenuation of samples to electromagnetic waves [16].

### 3.2. Emi Shielding Performance

An RSM model was developed to predict the total EMI shielding effectiveness (SET) as a function of three key parameters: material thickness (*d*, mm), G-MWCNT filler loading (*W*, wt%), and irradiation dose (*I*, kGy). The estimated regression coefficients of the fitted model Equation (8) are given in Table 2.

The regression coefficients, reported to seven decimal places for high precision, are intended to reflect the physical sensitivity of the shielding response to changes in the composite’s internal architecture, such as the density of the conductive network and the efficiency of interfacial bonding. This mathematical framework serves as the basis for the optimization objective, which is to maximize SET by identifying the synergistic interaction between thickness, filler loading and irradiation dose within the defined experimental design space. The utilized empirical approach was based on experimentally obtained data which was used as a data matrix for process modeling, and the established mathematical description of the studied system produced sufficient fit of the employed function.

The experimental plan was carefully designed to obtain sufficient data for process modeling purposes and potential interpolation within the investigated data range and in accordance with the established methodology. The regression coefficients are intended to reflect the physical sensitivity of the shielding response to changes in the composite’s internal architecture. Linear and interaction terms (such as *d*⋅*W*) represent how the density of the conductive network and the efficiency of interfacial bonding respond to variations in thickness and filler loading.

This approach uses an established mathematical description to provide a robust balance between fit quality (*R*^2^ = 0.96) and physical interpretability, ensuring the model is a meaningful representation of the studied system.

**Figure 8:**
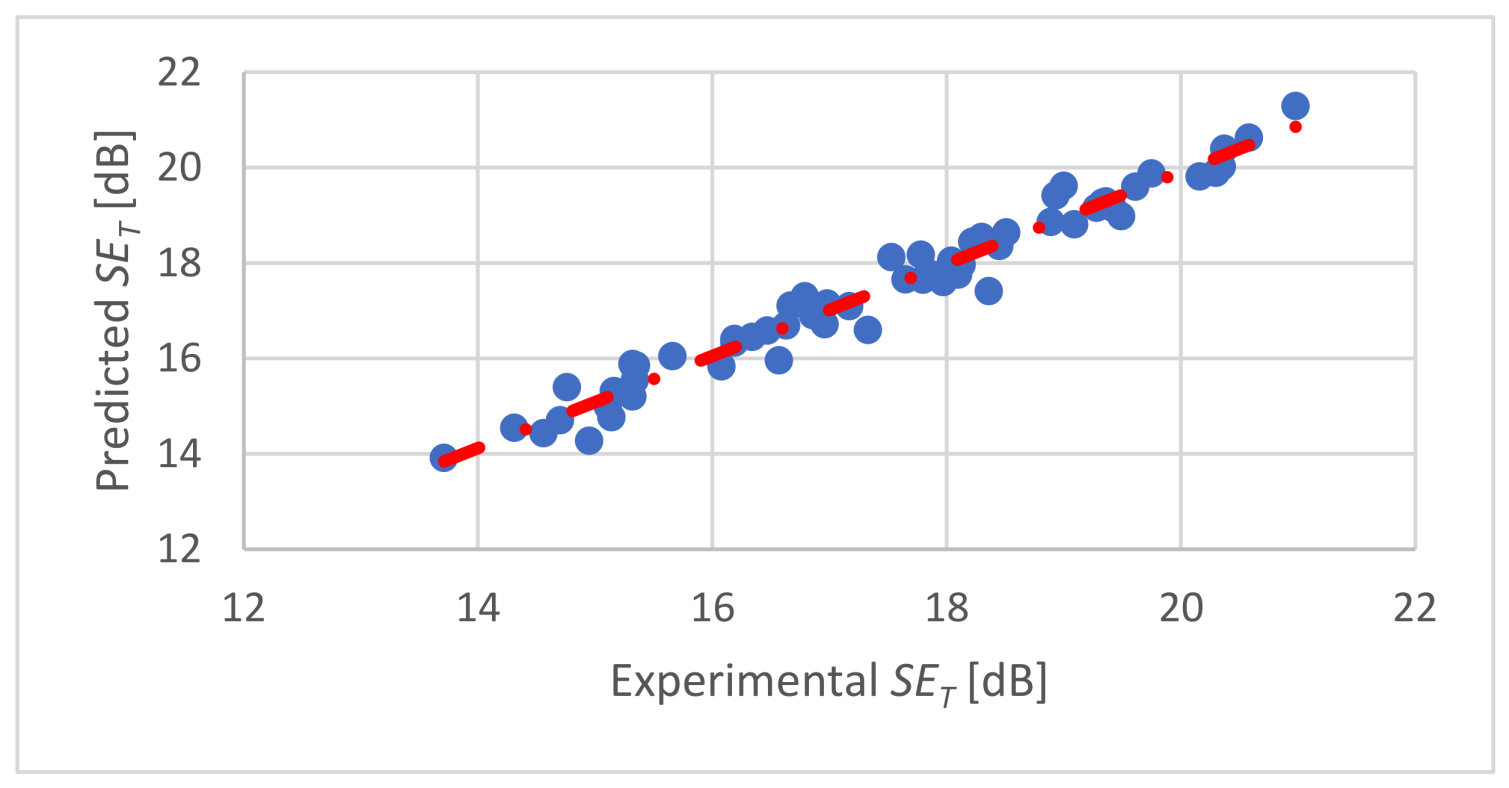
Predicted vs. experimental *SE_T_* values. The presented diagram suggests a good fit of model output values versus experimental data (blue dots) regarding the SE_T_ values in the investigated range, as it is confirmed by the correlation coefficient (*R*^2^ = 0.96) (red dashed line).

The presented quadratic model has a good fitting property and achieves excellent correlation with the experimental data (Figure 8), which provides a robust balance between fit quality and physical interpretability while minimizing overfitting risks. However, its predictive power outside the measured experimental range remains uncertain.

The presented figure clearly demonstrates that the mathematical model produces an appropriate fit for the experimental data, enabling further analysis of the investigated system.

A Response Surface Model (RSM) was developed to predict the total EMI shielding effectiveness (SET) using three key parameters: material thickness (*d*, mm), G-CNT filler loading (*W*, wt%), and irradiation dose (*I*, kGy). The obtained model produces a high correlation coefficient that corresponds to *R*^2^ = 0.96 and provides a good balance between fit quality and physical interpretability while minimizing overfitting risk. The initial interpretation of the influence of the studied process variables and significant interactions suggests that material thickness (*d*) and filler loading (*W*) have the strongest overall influence, particularly through their significant interaction (*d∙W*). The irradiation (*I*) produces a moderate contribution, while squared irradiation (*I*^2^) does not influence the EMI. In addition, interactions between thickness and material thickness (*I*∙*d*) and interactions between thickness and filler loading (*I*∙*W*) also have a moderate contribution.

The EMI shielding effectiveness of hybrid PMMA/G-MWCNT composites was evaluated in the S-band (2.65–3.90 GHz). Five samples with different filler loadings, thicknesses, and irradiation doses were tested. The key experimental results are summarized in Table 3.

Evidently, the predicted SE_T_ values derived from the model closely match the experimentally obtained data. For the samples containing hybrid nanostructures irradiated at 50 kGy but with varying hybrid contents (15 wt% and 20 wt%) and thicknesses (0.208 mm and 0.48 mm), higher SE_T_ values were recorded for the sample combining higher filler loading with greater thickness.

**Figure 9:**
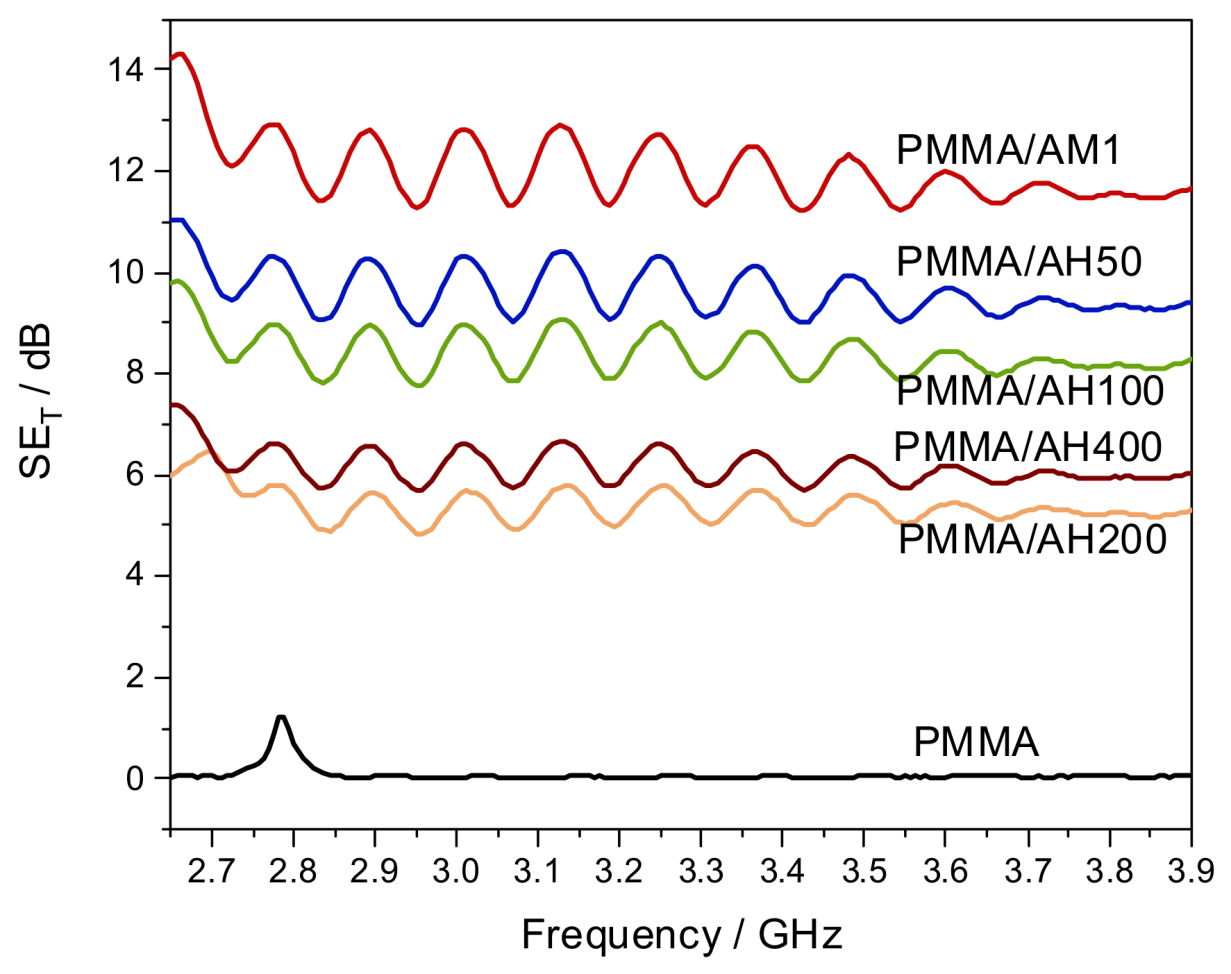
Total EMI shielding effectiveness (SE_T_) as a function of frequency for PMMA/AH and PMMA/AM1 nanocomposites and pure PMMA.

**Figure 10:**
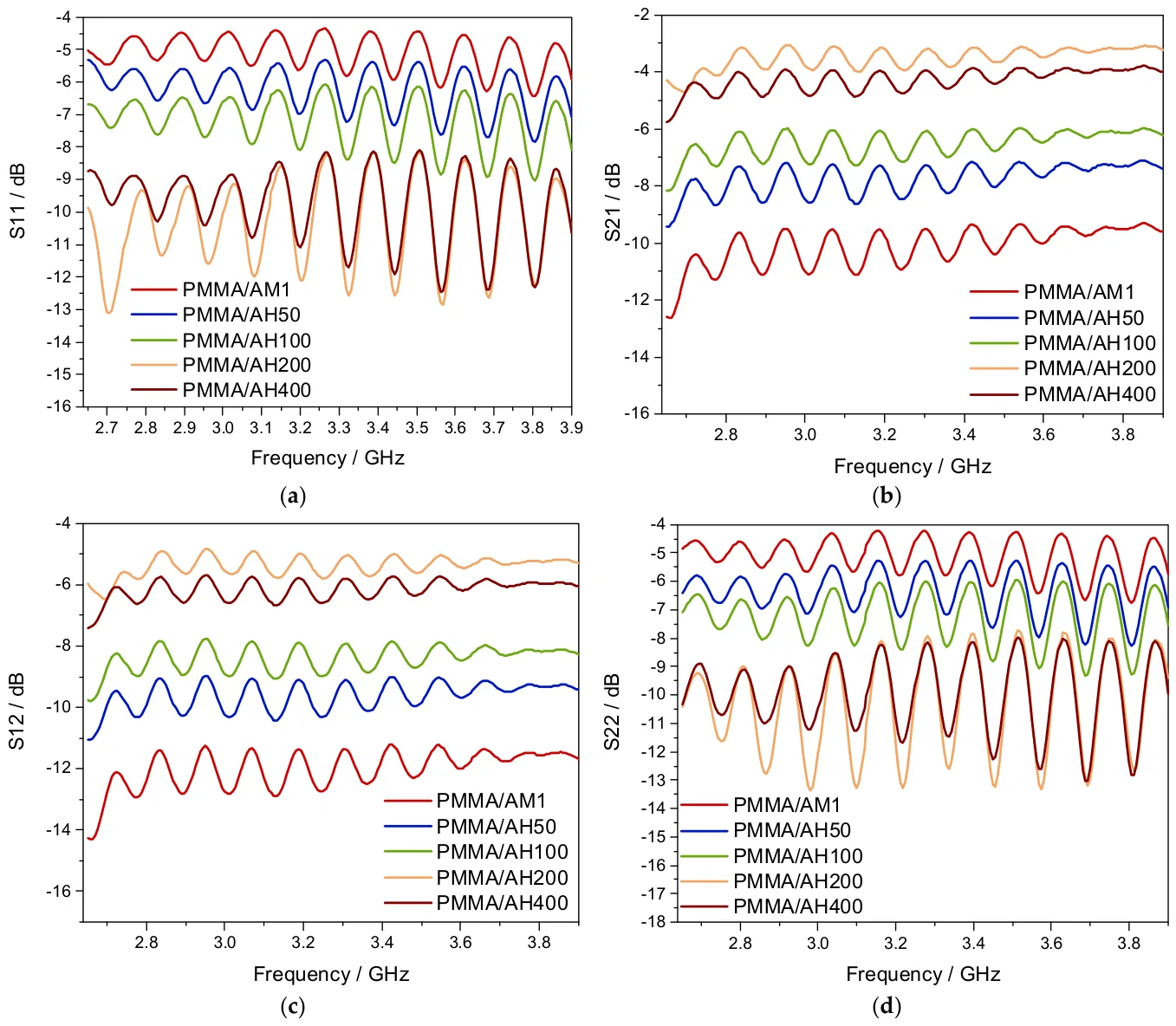
EMI SE real test measurements: (**a**) S_11_ scattering coefficient in dB, (**b**) S_21_ scattering coefficient in dB, (**c**) S_12_ scattering coefficient in dB, and (**d**) S_22_ scattering coefficient in dB, for the studied materials.

The experimental EMI SE measurements yielded the curves presented in Figure 9 and Figure 10. The irradiated PMMA/AM1 (50 kGy) sample (*d* = 0.48 mm) and the PMMA/AH50 sample (*d* = 0.28 mm) exhibited the highest shielding performance, reaching values of 11–14 dB. In contrast, the pure PMMA reference provided a shielding efficiency of less than 1 dB, confirming the critical role of the conductive hybrid G-MWCNT network.

As previously indicated by the morphological analysis, the nanocomposites containing G-MWCNTs irradiated at higher doses (100, 200, and 400 kGy) developed numerous internal nanopores, with an average diameter of 250–290 nm. These nanopores induced structural non-homogeneity within the bulk film, which ultimately resulted in the observed decrease in the total EMI SE. Furthermore, as illustrated in Figure 10, subtle variations between the forward (S_21_) and reverse (S_12_) transmission scattering parameters are present in certain irradiated samples. The single-sided irradiation and structural gradient do not break electromagnetic reciprocity in a passive linear material. Minor localized variations in contact impedance between the film surfaces and the WR-284 waveguide flanges also contribute to these minor transmission path differences.

**Figure 11:**
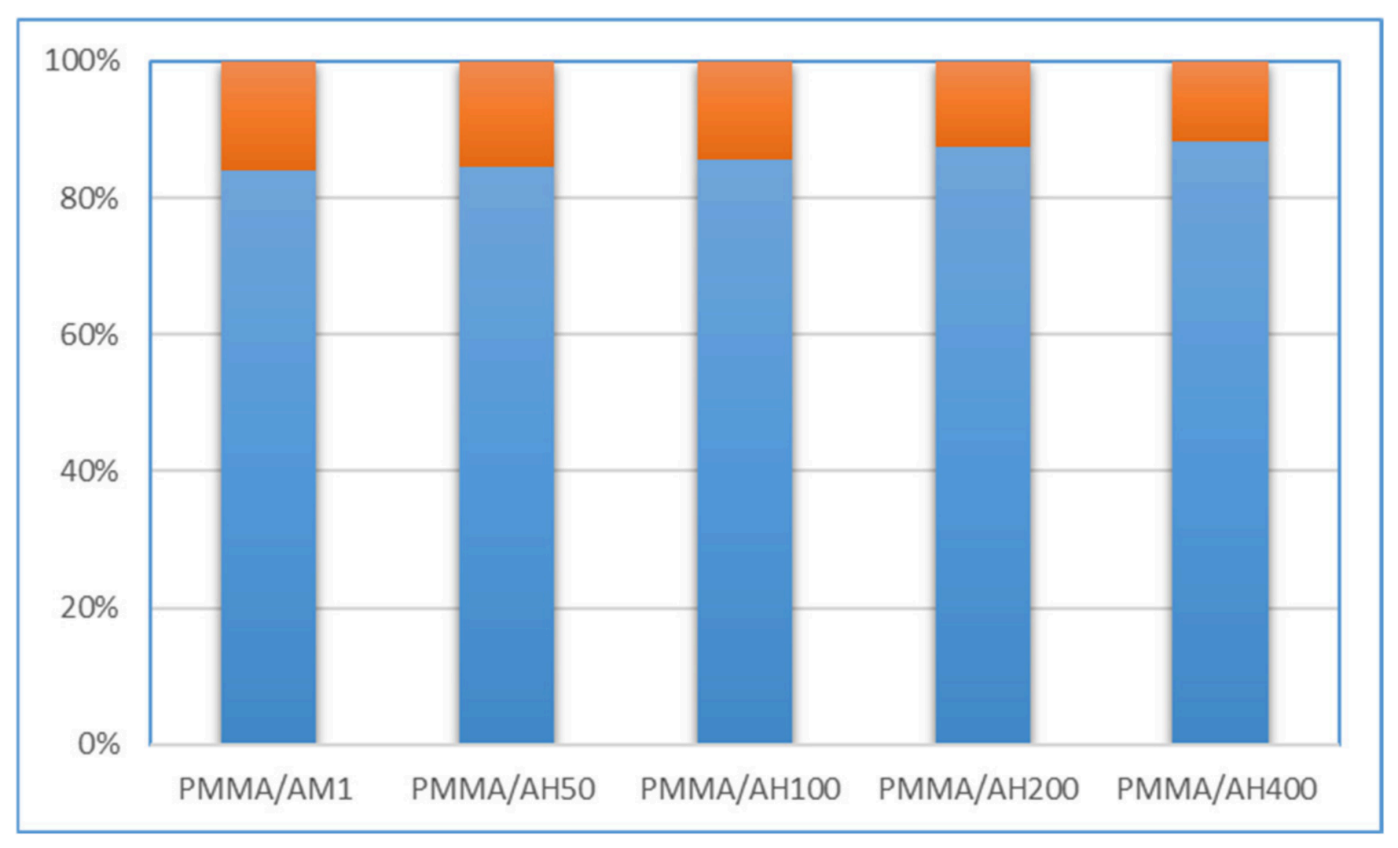
Contribution of absorption (SE_A_) (blue) and reflection (SE_R_) (orange) to total shielding effectiveness.

All prepared composites demonstrated an absorption-dominant shielding mechanism, with the absorption component (SE_A_) contributing 82–87% of the total SE_T_, as shown in Figure 11. The multiple reflection term (SE_M_) was calculated using the relationship SE_M_ = SE_T_ − SE_R_ − SE_A_ and was found to be mathematically negligible (<10^−10^ dB) across all samples and frequencies. This validates the standard approximation of SE_T_ ≈ SE_R_ + SE_A_ for these lightweight materials.

Jovanovic [7] and Pavlou [8] have published a state-of-the-art comparison of various EMI shielding data of graphene-based polymer nanocomposites. Comparison of our practically obtained data for carbon hybrid nanostructure with other graphene forms is very complex because there are different conditions, film thicknesses, frequency ranges, etc. However, comparison with other graphene-based nanocomposite films with similar thickness and filler loading has shown that our hybrid G-MWCNT nanostructures, compared to other graphene forms and nanostructures, exhibit lower EMI SE [7,8].

**Figure 12:**
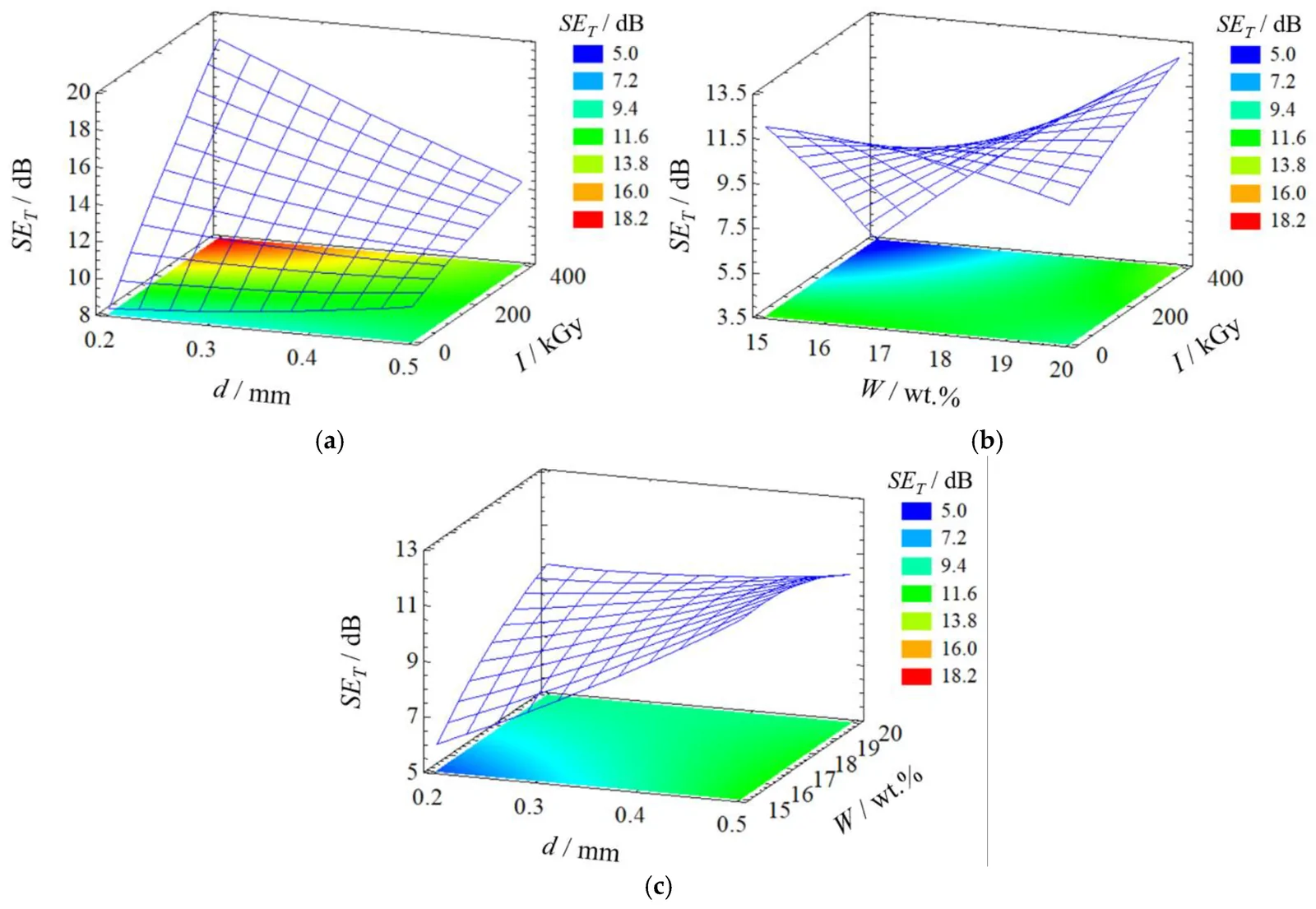
Three-dimensional response surface plots of predicted *SE_T_* as a function of (**a**) thickness (*d*) and irradiation (*I*) at fixed loading *W* = 20 wt.%, (**b**) loading (*W*) and irradiation (*I*) at a fixed thickness of *d* = 0.48 mm and (**c**) thickness (*d*) and loading (*W*) at fixed irradiation of *I* = 50 kGy.

Three-dimensional response surface plots (Figure 12) were generated based on the developed predictive model to visualize the functional dependence of the total EMI shielding effectiveness on the studied parameters and their interactions. The 3D response surfaces demonstrate that the two-factor interaction between the investigated independent variables has a significant impact on the SE_T_. The results indicate that the shielding effectiveness of the studied material strongly depends on the combination of the parameters, confirming the complexity of the studied system. This implies that, through precise optimization and combination of the parameters, a satisfactory electromagnetic interference shielding performance can be achieved within the frequency range of 2.65–3.90 GHz.

The response surfaces demonstrate the strong positive interaction between thickness and filler loading. Higher irradiation doses provide additional improvement, especially at greater thicknesses. The results confirm that increasing sample thickness and G-CNT content significantly enhances EMI shielding due to greater absorption volume and denser conductive networks. The positive effect of irradiation is attributed to improved filler dispersion and interfacial bonding. The reduced RSM model successfully captures these trends and provides a practical tool for material design optimization within the studied range.

## 4. Conclusions

This study successfully developed and characterized hybrid graphene–carbon nanotube (G-CNT)-reinforced PMMA composites for electromagnetic interference (EMI) shielding applications in the S-band (2.65–3.90 GHz). Five composites with varying G-MWCNT loading (15–20 wt%), sample thicknesses (0.208–0.48 mm), and irradiation doses (from 50 to 400 kGy) were fabricated and evaluated.

The composites demonstrated absorption-dominant shielding behavior, with *SE_A_* contributing over 82% of the total shielding effectiveness (SE_T_). The highest performance was achieved by the 20 wt% irradiated sample (0.48 mm thickness), reaching an average SE_T_ of 10.13 dB, while the AH200 15 wt% sample (0.28 mm) exhibited the strongest overall shielding among the 15 wt% series, averaging 3.5 dB among predicted and measured values. The multiple reflection term (SE_M_) was found to be mathematically negligible (<10^−10^ dB), validating the conventional SE_T_ ≈ SE_R_ + SE_A_ approach for these materials.

The highest shielding efficiency was achieved by the PMMA/AM1 sample (20 wt% filler, 0.48 mm thickness, irradiated at 50 kGy), reaching an average SE_T_ of 10.13 dB. Within the 15 wt% series (AH), the 50 kGy sample yielded the maximum performance (SE_T_ = 7.75 dB at 0.28 mm). Higher e-beam doses (100–400 kGy) induced internal nanopores (250–290 nm) and structural degradation, which disrupted the conductive network and lowered total shielding performance.

A reduced, three-factor Response Surface Methodology (RSM) model was developed using thickness (*d*), filler loading (*W*), and irradiation dose (*I*) as input variables. The model showed excellent correlation with experimental data (*R*^2^ = 0.96) and revealed a strong synergistic interaction between thickness and filler loading. The model equation provides a valuable predictive tool for optimizing composite design within the studied range.

The developed irradiated G-MWCNT/PMMA composites combine light weight, simple solution-cast processability, and effective absorption-dominant shielding, making them promising candidates for thin, flexible EMI shielding components in modern wireless electronics and sub-6 GHz 5G applications.

## Figures and Tables

**Table 1:** Conductivity of PMMA/G-MWCNT hybrid nanocomposites.

Sample	*d*_film_ [mm]	Conductivity [S/m]
PMMA	0.190	2.3 × 10^−13^
PMMA/AH50	0.280	0.04180
PMMA/AH100	0.208	0.00813
PMMA/AH200	0.280	0.00106
PMMA/AH400	0.210	0.09020

**Table 2:** Model coefficients of the quadratic model for EMI shielding effectiveness.

Model Coefficient	E Values
$\beta_{0}$	−34.3318
$\beta_{1}$	63.0837
$\beta_{2}$	3.49155
$\beta_{3}$	−0.0748732
$\beta_{4}$	9.81473
$\beta_{5}$	−3.23117
$\beta_{6}$	−0.0649753
$\beta_{7}$	−0.0682255
$\beta_{8}$	0.00579882
$\beta_{9}$	−0.00000501632

**Table 3:** Experimental vs. predicted SET values using the reduced RSM model.

Sample ID	*d* (mm)	*W* (wt%)	*I* (kGy)	Experimental Avg. SE_T_ (dB)	Predicted SE_T_ (dB)
PMMA/AM1-50 (20% G-MWCNT)	0.48	20	50	10.13	9.87
PMMA/AH50 (15% G-MWCNT)	0.28	15	50	7.75	7.68
PMMA/AH100 (15% G-MWCNT)	0.208	15	100	6.52	6.71
PMMA/AH200 (15% G-MWCNT)	0.28	15	200	3.51	3.62
PMMA/AH400 (15% G-MWCNT)	0.21	15	400	4.28	4.19

## Data Availability

The original contributions presented in this study are included in the article. Further inquiries can be directed to the corresponding author.

## References

[1] Deshmukh K., Kovarík T., Punetha V.D., Pandey M., Pasha S.K.K., Rani P., Sadasivuni K.K. (2026). Graphene and its derivatives based polymer nanocomposites for electromagnetic interference shielding applications: A comprehensive review. Mater. Today Sustain..

[2] Sharma G.K., Pilla S., Raji S., Suresh R., James N.R., Rahees M., Prabhakaran K. (2026). Carbon composites for electromagnetic interference shielding: Progress, challenges and perspective. Carbon.

[3] Wu N., Hu Q., Wei R., Mai X., Naik N., Pan D., Guo Z., Shi Z. (2021). Review on the electro-magnetic interference shielding properties of carbon-based materials and their novel composites: Recent progress, challenges and prospects. Carbon.

[4] Mikinka E., Siwak M. (2021). Recent advances in electromagnetic interference shielding properties of carbon-fibre-reinforced polymer composites—A topical review. J. Mater. Sci. Mater. Electron..

[5] Suchea M., Tudose I.V., Pascariu P., Koudoumas E., Joseph K., Wilson R., George G. (2020). Chapter 12—Carbon-Based Nanocomposites for EMI Shielding: Recent Advances.

[6] Grozdanov A., Paunovic P., Dimitrievska I., Proseva M., Gorgieva A., Castaldo R., Gentile G. (2026). Structural, Thermal, and Electrical Characteristics of various Graphene/PMMA nanocomposites aimed for EMI shielding. J. Nanotechnol..

[7] Jovanović S., Huskić M., Kepić D., Yasir M., Haddadi K. (2023). A review on graphene and graphene composites for application in electromagnetic shielding. Graphene 2D Mater..

[8] Pavlou C., Koral C., Pastore Carbone M.G., Papari G., Andreone A., Manikas A.C., Galiotis C. (2021). Effective EMI shielding behaviour of thin graphene/PMMA nanolaminates in the THz range. Nat. Commun..

[9] Anderson L., Govindaraj P., Anga A., Mirabedini A., Hameed N. (2021). Modelling, fabrication and characterization of graphene/polymer nanocomposites for electromagnetic interference shielding applications. Carbon Trends.

[10] Xia X., Liu Y., Pan Y., Zhong Z. (2023). Multi-objective optimal design of high-efficient EMI shielding in porous graphene-reinforced nanocomposites. Int. J. Mech. Mater. Des..

[11] Prosheva M., Ozmen-Monkul B., Gumus G., Taskin D.K., Trajcheva A., Blazevska-Gilev J., Tomovska R. (2025). Waterborne advanced optical coatings based on polymer composite with hybrid graphene-metal phthalocyanine nanofiller. Prog. Org. Coat..

[12] Kim S.-I., Kim J.S., Moon J.Y., Hyeong S.K., Ghods S., Choi J.H., Kim D., Park D.S., Heo K., Lee J.H. (2023). Electromagnetic interference shielding of graphene/PMMA composites depending on growth temperature of CVD-graphene. Synth. Met..

[13] Balamurugan A., Kannan S., Selvaraj V., Rajeswari S. (2004). Development and Spectral Characterization of Poly(Methyl Methacrylate)/Hydroxyapatite Composite for Biomedical Applications. Trends Biomater. Artif. Organs.

[14] Rajabi M., Mahanpoor K., Moradi O. (2019). Preparation of PMMA/GO and PMMA/GO-Fe_3_O_4_ nanocomposites for malachite green dye adsorption: Kinetic and thermodynamic studies. Compos. Part B.

[15] Kadhim M.A., Al-Bermany E. (2020). Enhance the Electrical Properties of the Novel Fabricated PMMA-PVA/Graphene Based Nanocomposites. J. Green Eng..

[16] Tao J., Zhou J., Yao Z., Jiao Z., Wei B., Tan R., Li Z. (2021). Multi-shell hollow porous carbon nanoparticles with excellent microwave absorption properties. Carbon.

